# Supplementary material for: Chromothripsis during telomere crisis is independent of NHEJ, and consistent with a replicative origin
Source: Genome Res. 2019 May;29(5):737–49. doi: 10.1101/gr.240705.118 (PMC6499312; doi:10.1101/gr.240705.118)
Supplement: Supplemental Material [file supp_gr.240705.118_Supplemental_file_1.zip › contigs/annotated_contigs/DB51/contig.2.DB51_length_521_mean_cov_6.26103646833.docx]

**DB51_length_521_mean_cov_6.26103646833**

```
ACAAGTGACATCTGAAAAGGTGATTTCAAATGAAGCATTTCTCAATCCAAATCAGATAGGAAATTAAGGTATAATTATTTGATAAGATA
 >chr4:177650277-177650430 + E=2e-80
AAGAATGTTTAAGTTATTAAACAGATATAGTATGTAAAATATAGCCATCTGTTTTAAAAATTTT|AAAGACAATAAAGTACGAAATTAT
                                                                  >chr4:177653256-177653
TTGCAATATACATAATGGATAAAAATGGACTAATATTCTTAAAACACTGCATGAATACTGGTGCTTCAATAGAAAGATGGACAAAGAAG
624 + E=6e-204
GTGAACTGAATATTAACGAAGAAGAAATATAAATAATACATATTGTATGATGGCAAAAGTGAAAGAAACTATAATTCTTTTCAATCCTT

TTCAATCTATATCTAAGAGACCCCCAGACTAAAGTTCAAGTTCATTCTTTTAACAAATTAGCACCTGAAGTTTTCTTGCATTTGTAGCA

ATTAGCACCTAATTTGTTAAAAGAATGAACTTGCATTTTAATCTGGGGGTCTCCTAGGTACAGATTGGTTTGAGGAA
```
